# Tibiotalar and Tibiotalocalcaneal Arthrodesis with Paragon28 Silverback^TM^ Plating System in Patients with Severe Ankle and Hindfoot Deformity

**DOI:** 10.3390/medicina59020344

**Published:** 2023-02-11

**Authors:** Carlo Perisano, Adriano Cannella, Chiara Polichetti, Antonio Mascio, Chiara Comisi, Vincenzo De Santis, Silvio Caravelli, Massimiliano Mosca, Giorgio Alfredo Spedicato, Giulio Maccauro, Tommaso Greco

**Affiliations:** 1 Department of Ageing, Neurosciences, Head-Neck and Orthopedics Sciences, Orthopedics and Trauma Surgery Unit, Fondazione Policlinico Universitario Agostino Gemelli IRCCS, 00168 Rome, Italy; 2 Orthopedics and Trauma Surgery, Mater Olbia Hospital, 07026 Olbia, Italy; 3 U.O.C. II Clinic of Orthopedics and Traumatology, IRCCS Istituto Ortopedico Rizzoli, 40136 Bologna, Italy; 4 Department of Statistics and Quantitative Methods, University of Milano-Bicocca, 20126 Milan, Italy

**Keywords:** ankle arthrodesis, ankle fusion, end-stage ankle osteoarthritis, tibiotalar fusion, tibiotalocalcaneal arthrodesis, total ankle replacement

## Abstract

Background and Objectives: The treatment of end-stage ankle osteoarthritis (OA) and associated hindfoot deformities remains a major challenge for orthopedic surgeons. Numerous techniques and surgical approaches have been proposed for tibiotalar (TT) and tibiotalocalcaneal (TTC) arthrodesis, from arthroscopic to open, as well as numerous devices proposed for internal fixation (retrograde intramedullary nails, cannulated screws, and plating systems). The aim of this study was to retrospectively analyze the results, with at least 18 months of follow-up, with Silverback^TM^ TT/TTC Plating System Paragon28 in a group of 20 patients with severe OA and hindfoot deformities (mainly secondary post-traumatic OA). Materials and Methods: The demographic characteristics and past medical history of the patients were collected and analyzed to identify the cause of the pathology. The degree of OA and deformity were quantified based on foot and ankle weight-bearing radiography and CT examination. Pre- and post-operative clinical and functional scores (ROM, VAS, AOFAS, FFI, and SF-36) and radiographic parameters (anterior distal tibial angle, tibiotalar angle, coronal tibiotalar angle, and hindfoot alignment angle) were evaluated. Results: All of the patients showed clinical and radiographic fusion at an average of 14 weeks (range 12–48), with improvement in pain and functional scores, without major surgical complications and/or infections. Conclusions: Despite the limitations of our study, the results with this new plating system showed good results in terms of bone consolidation, post-operative complications, and improvement of pain and quality of life in patients with severe OA and deformities of the ankle and hindfoot.

## 1. Introduction

Ankle osteoarthritis (OA), involving the tibiotalar (TT) joint, affects about 1% of the adult population with resultant functional limitations comparable to end-stage renal disease and congestive heart failure [1,2]. Ankle OA has remained as the main indication for TT arthrodesis or fusion since its first description in 1882 [3], especially in the end stages which can alter physiological gait leading to the development of subtalar (ST) joint degeneration with OA. When both joints (TT and ST) are severely affected, the surgical indication often shifts to tibio–talo–calcaneal (TTC) arthrodesis [4]. In addition to primary OA, other causes described in the literature may lead to TT or TTC arthrodesis surgery including post-traumatic OA [5], failures after total ankle replacement (TAR) [6], Charcot neuroarthropathy of the ankle [7], avascular necrosis (AVN) of the talus [8], inflammatory arthropathy [9] and bone loss due to bone tumors [10], or septic arthritis [11,12]. Different surgical techniques for TT and TTC arthrodesis have been described in the literature combining different surgical approaches and fixation hardware (internal or external) [8,13,14]. To prepare the joint surfaces for TT or TTC arthrodesis, both open and arthroscopic approaches can be used. Open approaches have been described as anterior, lateral, or posterior [4,15,16,17], while the arthroscopic approach is based on two portals (antero-medial and antero-lateral or postero-medial and postero-lateral) [18,19]. A wide variety of fixation devices such as cannulated screws [20,21,22], plates and screws [4,23,24], retrograde intramedullary nails [8,19,25], and external fixators [26,27,28] have been used amongst the various approaches.

Advances in surgical techniques and the development of new fixation devices have led to a lowering of the non-union rate in TT [13,16,24] and TTC [29,30] arthrodesis by up to 10% compared to historical case histories that also reported non-union rates of up to 40% [31].

Regardless of the surgical approaches and fixation devices used, the followings are essential for a good result: careful soft tissue management (especially in chronic vasculopathies or post-traumatic conditions) and joint surfaces’ debridement and preparation with good exposition of cancellous bone [31,32], good fit of the opposing bone surfaces with the right compression degree, and correct hindfoot alignment (neutral dorsiflexion, 5° hindfoot valgus, 5 to 10° external rotation, and the absence of antero-posterior translation) [5].

The aim of this study was to retrospectively analyze the clinical, functional, and radiographic results, with at least 18 months of follow-up, with the Ankle Fusion Silverback^TM^ TT/TTC Plating System (Paragon28, Englewood, CO, USA) in a group of 20 patients with severe TT and ST joint OA and hindfoot deformities.

## 2. Materials and Methods

### 2.1. Study Patients

A retrospective, observational study was conducted according to the STROBE guidelines [33].

All patients treated at the Department of Orthopedics and Trauma Surgery at our Institution between January 2016 and August 2020 with TT or TTC arthrodesis using the Ankle Fusion Silverback^TM^ TT/TTC Plating System (Paragon28, Englewood, CO, USA) [34] were included in the study.

The inclusion criteria were patients with primary or secondary TT joint OA, associated or not with ST joint OA, and with at least 18 months of follow-up after surgery. The exclusion criteria were TT or TTC arthrodesis associated with the fusion of other adjacent foot joints and patients with a follow-up of less than 18 months. Demographic characteristics, clinical features, and surgical data of the patients were collected retrospectively through medical and outpatient records.

All of the procedures were performed with the patients’ written informed consent and in accordance with institutional and national research committee ethical standards and the 1964 Declaration of Helsinki. The patients were not required to give informed consent for data processing for the study as the analysis used anonymous clinical data. The study gave notice to, was discussed with, and was approved by the board of the Department of Orthopedics and our school board.

### 2.2. Pre-Operative Management

All of the patients came to the outpatient clinic complaining of pain and functional impairment of the ankle and hindfoot due to end-stage OA of the TT joint, associated or not with ST joint OA. A careful analysis of the patients’ clinical history was conducted to identify the patients’ demographic characteristics, causes of deformity and OA, and any previous foot and ankle surgery. The clinical evaluation was completed with the following scores: range of motion (ROM) of the TT joint (normal value: 3° to 33° for dorsiflexion and 23° to 56° for plantarflexion) [35], the visual analogue scale (VAS) [36], the American Orthopaedic Foot and Ankle Society ankle–hindfoot scale (AOFAS) [37], the foot function index (FFI) [38], and the Short Form Health Survey 36 (SF-36) [39].

For pre-operative planning, foot and ankle standing radiography (in antero-posterior, lateral, and Saltzman views) and a computed tomography (CT) scan were obtained. Based on the imaging, OA was classified according to the Takakura classification [40] and the following angles were measured:

- Anterior distal tibial angle (ADTA), formed by the mechanical axis of the tibia and the TT joint orientation line in the sagittal plane (normal value: 80° ± 3°) [41];
- Tibiotalar angle (TTA), defined by the tibial and talar articular surfaces in the TT joint (if it measures >10°, the joint is defined as incongruent) [41];
- Coronal (or frontal) tibiotalar angle (CTTA), the superomedial angle between the longitudinal axis of the tibia, created by connecting two points in the middle of the proximal and the distal tibial shaft and the axis of the talus, defined by a line drawn through the talar shoulders in the anteroom-posterior view (normal value: 88.7° ± 5.1°) [42];
- Hindfoot alignment angle (HAA), formed by the intersection of the longitudinal axis of the tibial shaft and the axis of the calcaneal tuberosity (normal value: 5.6° ± 5.4°) [43].

### 2.3. Surgical Technique

The surgeries were performed in the supine position under general or peripheral anesthesia after the administration of antibiotic prophylaxis. Before skin preparation and draping, a pneumatic tourniquet was applied to the thigh and inflated just before the surgical incision.

An anterior or lateral approach to the TT join was chosen, depending on the local skin condition and any previous surgery performed. For the lateral approach, an osteotomy of the fibula approximately 6 cm from the apex of the peroneal malleolus was used. After soft tissue dissection, the TT joint was visualized and osteophytes were removed. A lamina spreader was used to fully expose and distract the joint so that careful debridement and preparation of the joint surfaces could be carried out until a subchondral cancellous bone was obtained, with osteotome and curettes rather than a bone saw or burrs to minimize the risk of thermal necrosis of the subchondral bone. In the case of particularly sclerotic bone, micro-perforations were performed with a fine awl. Once congruent joint surfaces had been obtained, under fluoroscopic control, the TT joint was temporarily fixed with 2 K-wires in neutral dorsiflexion, slightly in external rotation and in valgus (0–5°), with the talus reduced to a posterior position to obtain the largest possible contact area of the joint surfaces.

For the arthrodesis of the ST joint, a lateral approach was used. An oblique incision along the tarsal sinus was performed for patients who had already received an anterior approach to the TT joint while the incision reaching the apex of the peroneal malleolus was prolonged towards the base of the 4th metatarsal for patients who had already received a lateral approach for TT joint preparation. With the aid of a lamina spreader, the whole joint cartilage was removed (approximately 2 mm) with a chisel and osteotome until congruent joint surfaces with cancellous subchondral bone were obtained.

For internal fixation of the arthrodesis, the Ankle Fusion Silverback^TM^ TT/TTC Plating System in type II anodized titanium alloy was chosen, which provides a wear-resistant surface and helps prevent seizing or friction between sliding titanium surfaces. This plating system was chosen mainly because of its low profile (1.5–2.0 mm) and its anatomical shape, which allows it to be applied anteriorly, posteriorly, or laterally, leaving ample flexibility in choosing the best surgical approach in patients with poor soft tissue conditions and in some cases, with previous surgery. Depending on the quality of the bone, a choice was made between locking or non-locking screws (with five possible diameters: 3.5, 4.2, 4.5, 4.7, or 5.2 mm) and the use of free compression cannulated screws through the precision guide [34]. For proximal fixation of the plate (in the tibia), 4.7 or 5.2 mm screws were used; in the talus, 3.5 or 4.2 mm screws were used; and for fixation in the calcaneus, 4.5 or 5.2 mm screws were used.

### 2.4. Post-Operative Management

After surgery, a compression bandage was applied and the ankle was placed in a neutral position in a short leg brace. Two weeks after surgery, the wound was examined and the sutures were removed; the patients were given advice to partial weight-bear with crutches for a further 2 weeks. Follow-up was performed by outpatient clinical and radiographic evaluation at 1, 3, 6, 12, 18, and 24 months after surgery. The indication on weight-bearing was given based on clinical and radiographic evaluation, but all of the patients achieved total weight-bearing within 6 months after surgery.

During the follow-up, primary outcomes in terms of pain relief (VAS), recovery of function (ROM, AOFAS, FFI, and SF-36), and radiographic assessment (in terms of bone union, changes in angles assessed pre-operatively, and adjacent joints OA) were evaluated independently by two of the authors (T.G. and C.P.—Carlo Perisano). Bone union was assessed radiographically by observing the presence of trabecular lines between the tibia and talus at the point of contact and the disappearance of the radiolucent line [44]. Adjacent joint arthritis was defined as the appearance of joint space narrowing or osteophyte formation on standing foot and ankle radiographs.

The secondary outcomes were the presence of surgical complications and the need for revision surgery.

### 2.5. Statistical Analyses

IBM SPSS Statistics 25 (New York, NY, USA) was used for the statistical analysis. Qualitative data were expressed as numbers (with percentages) and quantitative data were expressed as means (± standard deviation or range). The Shapiro–Wilk test was used to determine the normal distribution of data. A paired t-test was performed to evaluate the difference between the pre-operative and post-operative values for normally distributed variables and a Wilcoxon test was used for non-normally distributed variables. The correlation between the variables was examined using Spearman correlation coefficients. Correlation was considered poor if r was <0.3, moderate if r was >0.3 and <0.6, good if r was >0.6 and <0.8, and excellent if r was >0.8. A *p*-value of <0.05 was considered statistically significant.

## 3. Results

The demographic characteristics of the patients are shown in Table 1; 20 patients were included, 13 males and 7 females, with a mean age of 60.0 years (range 40–74). The mean follow-up was 21 months (range 18–29).

**Figure 1:**
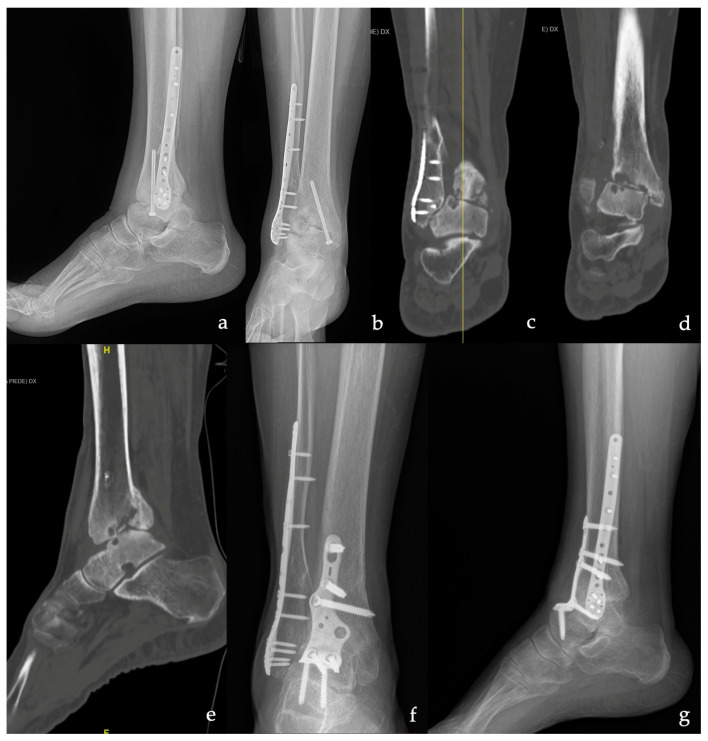
A 74-year-old woman with secondary post-traumatic TT osteoarthritis (OA). (**a**,**b**) Pre-operative X-ray in the lateral and anteroposterior view. (**c**,**d**) Coronal pre-operative CT scan showing severe OA of the TT joint. (**e**) Sagittal pre-operative CT scan at the level of the yellow line in figure (**c**). (**f**,**g**) Anteroposterior and lateral X-ray at 6 months of follow-up from tibiotalar (TTC) arthrodesis with the anterior plate showing complete bone union.

**Figure 2:**
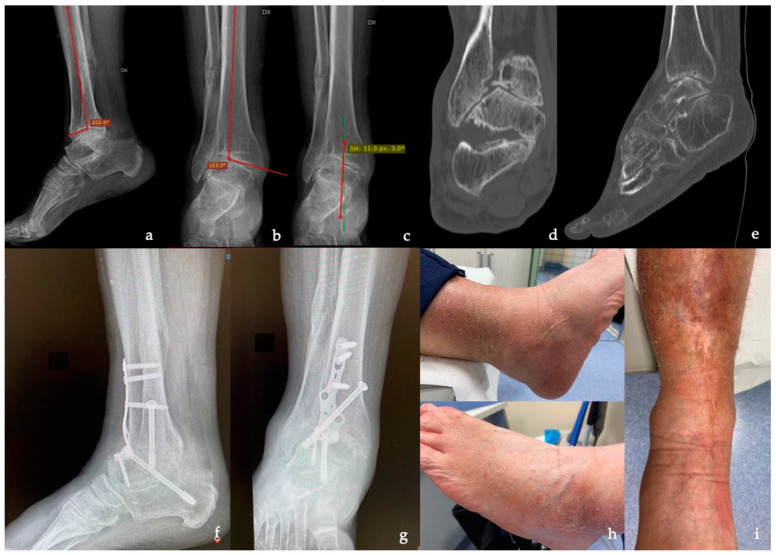
A 55-year-old man with severe osteoarthritis (OA) and deformity of the right ankle and a history of rheumatoid arthritis, diabetes mellitus, and venous insufficiency. (**a**–**c**) Pre-operative X-ray in the lateral and anteroposterior view. (**d**,**e**) Pre-operative CT showing severe OA of the TT joint. (**f**,**g**) Lateral and anteroposterior X-ray at 3 months of follow-up from tibiotalocalcaneal (TTC) arthrodesis with the anterior plate showing complete bone union. (**h**,**i**) Clinical image of the soft tissue condition.

**Figure 3:**
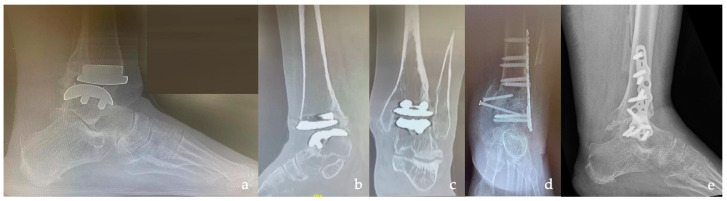
A 55-year-old man with severe osteoarthritis (OA) and deformity of the right ankle and a history of rheumatoid arthritis, diabetes mellitus, and venous insufficiency. (**a**–**c**) Pre-operative X-ray in the lateral and anteroposterior view. (**d**,**e**) Pre-operative CT scan showing severe OA of the TT joint.

In 11 patients, the ankle OA was post-traumatic (Figure 1), while in 4 patients, it was primary (Figure 2), in 2 cases, it was Charcot neuroarthropathy, in 2 cases, it was talus AVN, and in one case, it was due to aseptic loosening after TAR (Figure 3). Most of the patients had a severe grade of OA and nine patients showed grade 4 according to the Takakura classification.

The most frequent plate used for the fixation was the anterior (10 TT and 6 TTC arthrodesis), while in 4 cases, a lateral surgical approach with a lateral plate was used (3 TT and 1 TTC). The mean surgical time was 101.8 min (range 78–121).

Based on the post-operative clinical and radiographic evaluation, all of the patients (100%) achieved bone union within 12 months after surgery (Table 2). One patient, who was initially treated with a lateral plate, showed a delay in consolidation at 6 months; judging poor stability as the cause of the delay in consolidation, he was re-operated on with the addition of two cannulated screws in compression to promote bone union, which occurred within a further 3 months.

Regarding functional scores, there was a statistically significant improvement in VAS, AOFAS, FFI, and SF 36 values from pre-op to last follow-up (respectively VAS: 7.9 pre-op vs. 2.2 post-op, *p* = 0.002; AOFAS: 23.2 pre-op vs. 68.6 post-op, *p* = 0.002; FFI: 70 pre-op vs. 29 post-op, *p* = 0.002; SF-36: 29.2 pre-op vs. 51.9 post-op, *p* = 0.002).

Regarding the alignment of the ankle and hindfoot, all of the evaluated angles (ADTA, TTA, CTTA, and HAA), which had a pathological value before surgery, were in the physiological range post-operatively.

Two minor complications occurred requiring two secondary minor surgical treatments. One patient experienced delayed consolidation and underwent surgical treatment to add two cannulated screws in compression, while another patient underwent wound debridement surgery following wound dehiscence.

No patients had non-union, neurological injury, tendon injury, infection, rupture, or loosening of the fixation devices. In addition, no adjacent joint OA was found at the final follow-up.

The results of the correlation analysis between the post-op VAS, AOFAS, FFI, and Sf-36 parameters of the study participants are presented in Table 3 and the following statistically significant correlations were found: VAS post-op showed a negative (moderate strength) association with AOFAS post-op and SF-36 post-op, and AOFAS post-op showed a negative association with FFI post-op (excellent strength).

## 4. Discussion

End-stage ankle OA, regardless of its etiology, can reduce and severely limit patients’ quality of life like end-stage renal and cardiac failure, which is why proper management of OA is of crucial importance [2].

The patients included in this study had a mean age of 60.0 years, which is consistent with the data from the literature which show that TT and TTC arthrodesis are mainly performed in the sixth decade of life and are equally distributed between men and women [15,16,24,45]. Furthermore, our case history confirms secondary (post-traumatic) ankle OA as the main indication for TT arthrodesis [17,46,47].

In this cohort of patients, an indication was given for open TT or TTC arthrodesis surgery with plate fixation because of the advanced stage of OA (demonstrated by the Takakura classification) and a high degree of ankle and hindfoot deformity (demonstrated by the angle values calculated in the pre-operatively). In terms of OA and deformity, it is difficult to compare this case history with others because there are no other studies in the literature that report pre- and post-operative ADTA, TTA, CTTA, and HAA values or the grade of the Takakura classification.

Currently, similar bone union rates in TT arthrodesis with different fixation devices are reported in the literature; although, some studies report better results with plate fixation than with screw fixation alone [21] or with a retrograde nail [4].

Our bone union rate of 100% is consistent with outcomes reported in a recent systematic review by Van de Heuvel et al. (99%) [17]. The bone union time also does not differ greatly from the literature: 14 weeks on average in our study and other recent studies [6,16,17,48], slightly shorter than the 18 reported by Zhang et al. [4].

The arthroscopic approach causes less soft tissue damage, leading to earlier recovery with bone union rates not dissimilar to those in this study [6,18]; however, it should be used in patients with a malalignment of less than 15° and, due to the severe deformities and/or bone loss in our cohort, an open approach was chosen [18]. Furthermore, regarding arthroscopic TTC arthrodesis, only two cases successfully treated with retrograde nailing using two posterior portals for the preparation of both TT and ST joints are reported in the literature [19].

In our study, two out of twenty patients developed complications. The 5% superficial infection rate of our series is close to the 4.9% rate presented in van den Heuvel et al.’s systematic review [17], the 6.2% rate reported by Colman et al. [49], and the 6.89% rate of Mohamedan et al. [50]. The surgical revision rate of 5% in our study, due to the one case of delayed union at 6 months of a TTC plate, is like the 7.2% revision rate for non-union by Gharehdaghi et al. [47] and the non-union rate reported by the two systematic reviews [17,31] (10% and 4.5%, respectively). After the surgical procedures, our patients also showed an improvement in their clinical outcomes at 18 months in their AOFAS, VAS, SF 36, and FFI scores, with results (AOFAS 68.6) within the range of 67.5 to 76.2 reported by van Heuvel et al.’s review (AOFAS range 67.5–76.2) [17] and like other recent studies [4,16,48]. The improvement of the VAS scale and SF-36 are also in line with the results presented by Kim Park and Townshend [15,16,51], while the mean FFI score of 29% at the last follow-up is better than the 61.5% reported by Lowery et al. [52] in a retrospective study with at least 10 years of follow-up from 1999 to 2013. This discrepancy in the results could be related both to the different lengths of the follow-up periods and to improvements in surgical techniques and fixation hardware over the last three decades. The functional results at the last follow-up are also comparable to several recent TAR articles reporting a marked improvement in outcomes with AOFAS of 88.5 at 24 months [53] and FFI of 40.3 at a minimum follow-up of 6 years [52]. Despite these encouraging, new results, we chose to perform TT or TTC arthrodesis in these patients because of both the relatively low functional demands of the patients and the high risk of failure due to the poor bone quality, high deformity, and soft tissue conditions prior to surgery that characterized this study population.

The mean value of 101.8 min of operative time in our study appears longer than the 72.3 min reported by Zhang et al. [3], a difference justified by the long surgical times of the first operations when surgeons and operating room staff were developing familiarity with the new plating system. This also appears to be influenced by a prolonged duration in some patients in the case series, who had severe deformities and required more time for adequate preparation of the articular surfaces and proper realignment.

Based on these pre-operative conditions, the Silverback plating system was chosen, with it offering a wide range of anatomically shaped, low-profile plates (1.5 to 2.0 mm) and being specific for different surgical approaches (anterior, lateral, or posterior), with multiple fixation options in the tibia, talus, and calcaneus that, in combination with the guide precision system, allowed for adequate compression on the joint surfaces. This system, given the low profile of the hardware, allows for minimal damage and stress to the soft tissues, with greater patient tolerance.

The limitations of this study are the retrospective design on a relatively small population, without randomization and a control group. Instead, the medium–long-term follow-up and, above all, having selected a group of patients with severe deformity based on pre-operative evaluations (degree of OA and ankle alignment angles) can be considered strengths, which we believe they are necessary values to be reported in all studies to make the cases comparable.

## 5. Conclusions

Considering the medium–long-term follow-up and the high grade of deformity and OA of the patients before surgery, due to various causes, this study shows good and encouraging results in terms of clinical outcomes and patient satisfaction, allowing us to propose this plating system for TT or TTC arthrodesis. Nevertheless, no conclusive results can be drawn given the small sample size, but studies with larger populations and longer follow-ups will be necessary.

## Figures and Tables

**Table 1:** Demographic characteristics of the patients.

**Patients**	20	
**Sex (M-F)**	13-7	
**Age (Mean, Range)**	60.0 (40–74) years old	
**Causes of OA/Deformities**	Post-traumatic	11 (55%)
	Primary	4 (20%)
	CNO	2 (10%)
	Talus AVN	2 (10%)
	TAR aseptic loosening	1 (5%)
**Side (Left-Right)**	9–11	
Takakura Classification *	Grade 1	0 (0%)
	Grade 2	2 (10%)
	Grade 3a	4 (21%)
	Grade 3b	4 (21%)
	Grade 4	9 (48%)
**TT pre-op ROM (Mean, Range)**	43° (27–65)	
**Type of Plate**	Anterior TT	10 (50%)
	Anterior TTC	6 (30%)
	Lateral TT	3 (15%)
	Lateral TTC	1 (5%)
**Surgical Time (Mean, Range)**	101.8 (78–121) min	
**Follow-Up (Mean, Range)**	21 (18–29) months	

(* Based on 19 patients, excluding the patient with TAR aseptic loosening. M: male; F: female; OA: osteoarthritis; CNO: Charcot neuroarthropathy; AVN: avascular necrosis; TAR: total ankle replacement; TT: tibiotalar; TTC: tibiotalocalcaneal; ROM: range of motion.)

**Table 2:** Pre-operative and last follow-up mean values with standard deviation and ranges.

**Bone Union**	(20/20) 100%		
**Time of union** **(mean, range)**	14 (12—48) weeks		
**Complications**	One delayed consolidationOne wound dehiscence		
	**Pre-op**	Post-op *	***p*-value**
**VAS**	7.9 ± 1.4 (5–10)	2.2 ± 1.1 (1–4)	**0.002 ^§^**
**AOFAS**	23.2 ± 11.9 (7–44)	68.6 ± 8.9 (50–78)	**0.002 ^§^**
**FFI**	70 ± 13 (54–88)	29 ± 14 (15–50)	**0.002 ^§^**
**SF-36**	29.2 ± 3 (23–33)	51.9 ± 6.2 (42–60)	**0.002 ^§^**
**ADTA**	83.0° ± 12.0° (71–102) ^†^	77.8° ± 4.8° (65–82) ^‡^	-
**TTA**	23° ± 9.0° (17–38) ^†^	8 ± 2 (5–12) ^‡^	-
**CTTA**	97.5° ± 7° (90–108) ^†^	89.3° ± 2° (87–92) ^‡^	-
**HAA**	20° ± 5° (14–29) ^†^	9° ± 5° (5–13) ^‡^	-

(* At last follow-up. ^§^ Wilcoxon test, statistically significant for *p* < 0.05. ^†^ Pathological value; ^‡^ Normal value.VAS: visual analogue scale; AOFAS: American Orthopaedic Foot and Ankle Society ankle–hindfoot scale; FFI: foot function index; SF-36: Short Form Health Survey 36; ADTA: anterior distal tibial angle; TTA: tibiotalar angle; CTTA: coronal tibiotalar angle; HAA: hindfoot alignment angle.).

**Table 3:** A matrix of the Spearman correlation coefficient (rho) between VAS, AOFAS, FFI, and SF-36 post-op.

	VAS Post-Op	AOFAS Post-Op	FFI Post-Op	SF-36 Post-Op
**VAS post-op**	1			
**AOFAS post-op**	−0.47 *	1		
**FFI post-op**	0.31	−0.80 *	1	
**SF-36 post-op**	−0.49 *	0.25	0.13	1

(* Statistically significant for *p* < 0.05.VAS: visual analogue scale; AOFAS: American Orthopaedic Foot and Ankle Society ankle–hindfoot scale; FFI: foot function index; SF-36: Short Form Health Survey 36.)

## Data Availability

The study data will be available upon request to the corresponding author (email: greco.tommaso@outlook.it).
